# Ostéopoécilie associée à un rhumatisme psoriasique

**DOI:** 10.11604/pamj.2017.26.227.12213

**Published:** 2017-04-25

**Authors:** Zeineb Alaya, Walid Osman, Laassad Hassini, Houneida Zaghouani, Nader Naouar, Elyès Bouajina

**Affiliations:** 1 Service de Rhumatologie, CHU Farhat Hached, Sousse, Tunisie; 2 Service de Chirurgie Orthopédique, CHU Sahloul, Sousse, Tunisie; 3 Service d’Imagerie Médicale, CHU Farhat Hached, Sousse, Tunisie

**Keywords:** Ostéopoécilie, radiographie, diagnostic, psoriatic arthritis, Osteopecilia, radiograph, diagnosis, psoriatic arthritis

## Abstract

L'otéopoécilie est une ostéopathie condensante bénigne et rare. Son association à un rhumatisme inflammatoire est très rare. Nous en rapportons un cas. Patiente âgée de 25 ans, atteinte d’un psoriasis cutané, a consulté pour douleur inguinale inflammatoire. L’examen a montré une limitation des hanches, une inégalité de longueur des membres inférieurs et une douleur à la mobilisation de la sacro-iliaque droite. La biologie a montré un syndrome inflammatoire et un bilan immunologique négatif. La radiographie du bassin a révélé une ostéopoécilie associée à une coxite destructrice. La TDM du bassin a montré en plus de la coxite et de l'ostéopoécilie une sacro-illite bilatérale. Le diagnostic d’un rhumatisme psoriasique associé à une ostéopoécilie a été retenu. La patiente a été mise sous méthotrexate et AINS. L'ostéopoécilie est souvent de découverte fortuite. Son diagnostic radiologique est impératif afin d'éviter des explorations et des traitements inutiles.

## Abstract (en)

Osteopecilia is a benign and rare condensing osteopathy. Its association with inflammatory rheumatism is very rare. We here report the case of a 25-year old patient with skin psoriasis, presenting with groin pain of inflammatory origin. Physical examination showed limitation of hip motions, lower limb-length inequality and pain on right sacroiliac mobilization. Laboratory tests showed inflammatory syndrome and negative immunological assessment. The radiograph of the pelvis revealed osteopecilia associated with destructive coxitis. CT scan of the pelvis showed coxitis and osteopecilia associated with bilateral sacroiliitis. The diagnosis of psoriatic arthritis associated with osteopecilia was retained. The patient was treated with methotrexate and NSAIDS. Osteopecilia usually is unexpectedly detected. Diagnostic radiology is essential to avoid unnecessary explorations and treatments.

## Introduction

L'otéopoécilie est une ostéopathie condensante bénigne et rare [1]. Son association à un rhumatisme inflammatoire a été rarement rapporté [2]. Nous rapportons un cas d'association ostéopoécilie et rhumatisme psoriasique.

## Patient et observation

**Figure 1:**
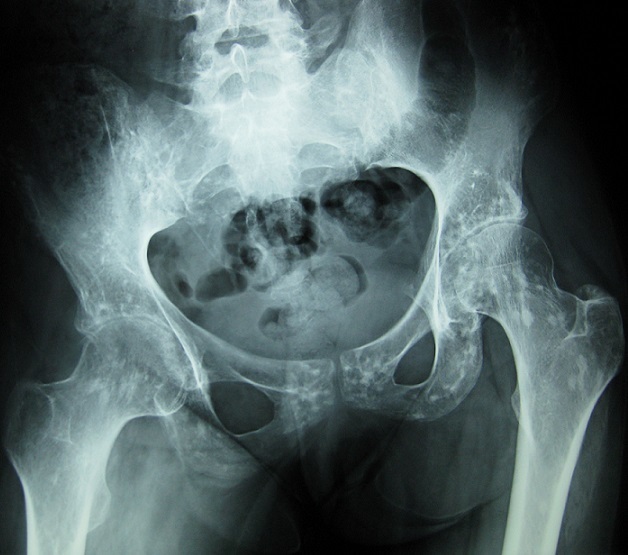
Radiographie standard du bassin révélant de multiples lésions condensantes arrondies ou ovoïdes symétriques siégeant sur les extrémités supérieures des fémurs, des cotyles, des branches ischio-pubiennes, de la symphyse pubienne, des ailes iliaques et du sacrum en rapport avec une ostéopoécilie associées à un pincement coxo-fémoral bilatéral avec érosions de la tête fémorale et du cotyle en rapport avec une coxite

**Figure 2:**
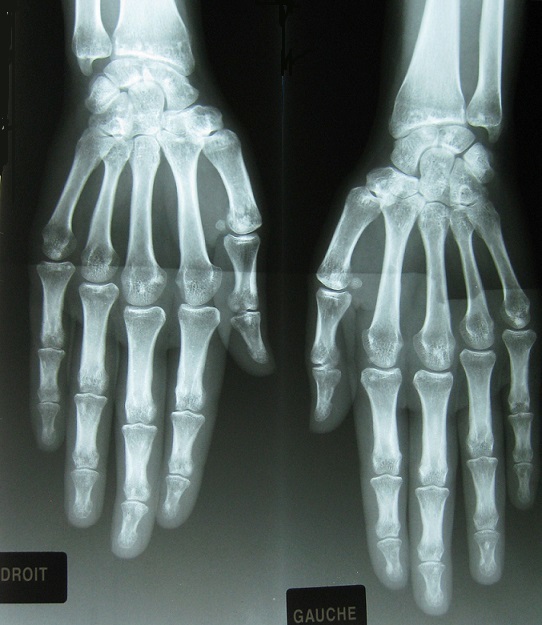
Radiographie des mains de face montrant des lésions denses intéressant les os du carpe, l’extrémité inférieure du radius et du cubitus bilatérales et symétriques en rapport avec une ostéopoécilie

**Figure 3:**
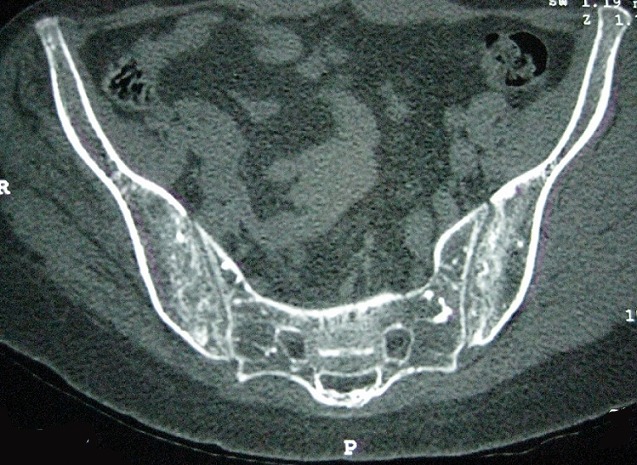
TDM du bassin montrant des érosions des berges des deux sacro-iliaques sans condensation en rapport avec une sacro-iliite bilatérale associée à une ostéopoécilie

Patiente âgée de 25 ans, atteinte d’un psoriasis cutané, a consulté pour douleur inguinale droite de type inflammatoire évoluant depuis 2 ans, sans autres signes associés. L’examen a mis en évidence une mobilisation limitée des deux hanches dans tous les plans, une inégalité de longueur des membres inférieurs, une douleur à la mobilisation de la sacro-iliaque droite et un signe de Trépied positif. La biologie a montré un syndrome inflammatoire avec une VS à 89 mm (H1) et un bilan immunologique (AAN, FR, Ac anti CCP) négatif. La radiographie standard du bassin (Figure 1) a révélé de multiples lésions condensantes arrondies ou ovoïdes symétriques siégeant sur les extrémités supérieures des fémurs, des cotyles, des branches ischio-pubiennes, de la symphyse pubienne, des ailes iliaques et du sacrum en rapport avec une ostéopoécilie associées à un pincement coxo-fémoral bilatéral avec érosions de la tête fémorale et du cotyle en rapport avec une coxite destructrice. La radiographie des mains de face (Figure 2) a montré des lésions denses intéressant les os du carpe, l'extrémité inférieure du radius et du cubitus bilatérales et symétriques en rapport avec une ostéopoécilie. La TDM du bassin (Figure 3) a montré des érosions des berges des deux sacro-iliaques sans condensation en rapport avec une sacro-iliite bilatérale associée à une ostéopoécilie. Le diagnostic d’un rhumatisme psoriasique associé à une ostéopoécilie a été retenu. La patiente a été mise sous méthotrexate et AINS.

## Discussion

L'otéopoécilie ou l’ostéosclérose familiale disséminée est une ostéopathie condensante bénigne et rare, généralement transmise selon un mode autosomique dominant à expressivité variable [1]. Sa pathogénie reste inconnue [2]. Classiquement, les sujets atteints sont asymptomatiques avec découverte fortuite de l'ostéopoécilie sur des radiographies, mais 15 à 20 % décrivent des douleurs et des épanchements articulaires [2]. L'image radiologique est typique, affirmant le diagnostic [1-3]. Il s'agit de lésions arrondies ou ovoïdes, multiples, bien limitées, homogènes, extrêmement denses, situées dans les régions osseuses périarticulaires [1-4]. L'atteinte est souvent symétrique prédominant sur les épiphyses et les métaphyses des os longs, du carpe, du tarse, du pelvis et des épaules; celle des côtes, des clavicules, des vertèbres et du crâne est plus rare et moins marquée [2-4]. La scintigraphie osseuse est généralement normale [2, 4]. La tomodensitométrie et l'IRM sont inutiles pour affirmer le diagnostic [4]. L’aspect radiographique, associé à un examen clinique normal, permet de poser le diagnostic [2-4]. Le diagnostic différentiel radiologique comprend surtout l’ostéopathie striée, la mélorhéostose, la sclérose tubéreuse, les métastases osseuses condensantes et l’ostéome [2, 4, 5]. Les complications sont exceptionnelles (sarcome, sténose du canal rachidien, fracture, ostéosclérose) et les cas rapportés peuvent traduire une association fortuite [2, 3]. L'ostéopoécilie n'augmente ni la morbidité ni la mortalité. Elle ne nécessite aucun traitement étiologique [2]. Elle peut être associée à d’autres anomalies comme des anomalies squelettiques, des malformations (coarctation aortique, double uretère), des dysfonctionnements endocriniens, des anomalies dentaires, faciales et à la dacryocystite [2]. Le plus souvent l'ostéopoécilie est isolée. Cependant, des lésions cutanées peuvent être observées dans 25% des cas, à type de dermatofibrose lenticulaire disséminée définissant le syndrome de Buschke-Ollendorf, mais également de chéloïdes ou de lésions sclérodermiformes. L'association à d'autres dysplasie fibreuses comme la mélorhéostose ou l'ostéopathie striée est possible, décrite sous le nom de “dystrophie osseuse sclérosante mixte” [4]. Concernant son association aux rhumatismes inflammatoires chroniques deux cas seulement ont été rapportés dans la littérature (polyarthrite rhumatoïde, spondyloarthrite séronégative) et pour ces deux cas l’association semble fortuite [2, 6].

## Conclusion

L'ostéopoécilie est une affection bénigne, souvent de découverte fortuite, dont le diagnostic radiologique est impératif afin d´éviter des explorations et des traitements inutiles. Son association à des rhumatismes inflammatoires chroniques est très rare.
